# Reproductive planning, vitamin knowledge and use, and lifestyle risks of women attending pregnancy care with a severe mental illness

**DOI:** 10.1080/02813432.2021.1882081

**Published:** 2021-02-11

**Authors:** Jacqueline Frayne, Yvonne Hauck, Thinh Nguyen, Helena Liira, Vera A. Morgan

**Affiliations:** a Medical School, Division of General practice, The University of Western Australia, Crawley, WA, Australia; b Department of Obstetrics, Women and Newborn Health Service, Subiaco, WA, Australia; c Department of Nursing and Midwifery Education and Research, Women and Newborn Health Service, Subiaco, WA, Australia; d Nursing, Midwifery and Paramedicine, Curtin University, Perth, WA, Australia; e Medical School, Division of Psychiatry, The University of Western Australia, Crawley, WA, Australia; f Peel and Rockingham Kwinana Mental Health Services, Rockingham, WA, Australia; g General Practice, University of Helsinki, Helsinki, Finland; h Neuropsychiatric Epidemiology Research Unit, School of Population and Global Health, The University of Western Australia, Crawley, WA, Australia; i Centre for Clinical Research in Neuropsychiatry, Medical School, Division of Psychiatry, The University of Western Australia, Crawley, WA, Australia

**Keywords:** Unintended pregnancy, mental disorders, prenatal vitamins, bipolar, schizophrenia, prevention

## Abstract

**Objective:**

Women with severe mental illnesses are a vulnerable population and little is known about their reproductive planning needs. The aim of our study was to describe rates of unintended pregnancies, postpartum contraception, identify use and knowledge of prenatal/pregnancy vitamins and identify modifiable lifestyle risks.

**Design:**

Mixed methods study incorporating a cross-sectional survey and prospective pregnancy data collection

**Setting:**

A multidisciplinary antenatal clinic in Australia

**Method:**

Thirty-eight pregnant women with severe mental illnesses: schizophrenia, schizoaffective, bipolar and severe post-traumatic stress disorder

**Main outcome measures:**

Unintended pregnancy rates, immediate postpartum contraception, use of prenatal and pregnancy vitamins and knowledge sources, obesity, and use and cessation rates for smoking, and substances, and comorbid medical conditions

**Results:**

Overall 42% of women had unintended pregnancy, with those with schizophrenia at most risk (56%). A long acting reversible contraception was inserted in 5 women (13%), with 45% having no immediate contraception prescribed prior to postnatal discharge. Women’s main source of vitamin supplementation for pregnancy was from general practitioners. Prenatal folic acid use occurred in 37%, with rates differing for those with a diagnosis of bipolar disorder (52%) and schizophrenia (25%). Vitamin deficiencies occurred in pregnancy, with iron deficiency (ferritin <30 ng/mL) (*n* = 27, 73%) the most frequent. Overall 21% of women smoked cigarettes and 35% were obese.

**Discussion:**

Addressing gaps in use of effective contraception, proactive reproductive planning and lifestyle management may improve outcomes for women with mental illnesses and their babies.

**Key points**
Women with severe mental illnesses have complex health needs that require targeted reproductive counselling. This study adds to what is known by highlighting that:
•Women with schizophrenia appear more likely to have unintended pregnancy.
•Prenatal counselling for women with severe mental disorders should include recognition and optimisation of management for the high rates of pre-existing medical comorbidities, obesity and elevated nicotine and substance use.
•Many women with severe mental illness need increased doses (5 mg) of prenatal folic acid due to psychotropic medication risk and obesity, as well as treatment for high rates of iron and vitamin D deficiency in pregnancy.

## Introduction

Health prior to pregnancy is recognised for its importance on pregnancy outcomes and the risk of development of non-communicable diseases in future generations [1]. Women with a severe or serious mental illnesses (SMI), often defined as ‘…a mental, behavioural, or emotional disorder resulting in serious functional impairment, which substantially interferes with or limits one or more major life activities’ [2] and most commonly in perinatal mental health referring to those with bipolar, schizophrenia and postpartum psychosis are significantly at risk.

General Practitioners (GPs) are integral to patient management and are ideally placed within the health care system to provide reproductive planning to those with existing or new mental health diagnoses. Women of reproductive age with an established SMI diagnosis appear to have satisfactory engagement with GPs in primary care [3]. Additionally, new diagnoses can be made in the peripartum period with research suggesting that women who develop postpartum psychosis visit the GP more frequently than healthy controls [4]. Ideally reproductive planning should be undertaken early, either prior to a pregnancy or in the postpartum period for subsequent pregnancies. Not only are women with SMI at increased psychiatric risk, requiring liaison with the treating psychiatric team, they have higher rates of medical and lifestyle comorbidities than those without mental illnesses, and their care is complicated by the use of psychotropic medications in pregnancy [5,6].

Current National Institute of Clinical Excellence (NICE) guidelines [7] recommend discussions with all women with mental illnesses around pregnancy planning and contraception use, especially given the lack of safety data and emerging evidence on benefits and risks of psychotropic medications use in pregnancy. Reproductive planning is, therefore, a vital strategy for the management of all women of child bearing age with SMI [6] and incorporates a health promotion approach, targeting modifiable lifestyle risks, managing chronic health concerns, as well as recommending vitamin supplementation in the optimisation of pregnancy care.

Within the general population, rates of unintended pregnancy are reported to occur in around 40% of instances [8], with higher rates suggested for women with chronic mental health diagnoses [3,9]. It has been reported that women with psychotic disorders are less likely to have discussions around contraception use [10] even though they appear to be more vulnerable due to high risk sexual practices, coerced sex and reduced use of contraception [3]. Therefore, delivering timely contraception advice to women with SMI who are of reproductive age [11] is essential to reduce the consequences from comorbid medical risks and unintended pregnancy. These include abortion, psychiatric deterioration, medication-induced teratogenicity, fetal exposure to nicotine, alcohol and substances, out of home child placement and long-term health effects for mother and child.

Despite this goal of reducing unintended pregnancies and its consequences, we know that many women with SMI fail to undertake prenatal counselling, even if they are taking a potential teratogenic medication such as lithium [12]. Women taking medications such as anticonvulsant mood stabilisers and those associated with fetal cardiac defects require higher dose folic acid supplementation [13,14]. Women exposed to obesogenic psychotropic medications may also require higher dose folic acid supplementation in the peripartum period given the recommendation for its use in obesity [15]. Overall many women in general remain uninformed of the need for prenatal care [16], therefore raising awareness of the importance of prepregnancy planning, particularly for women with SMI, may improve outcomes.

To identify needs and potential gaps in care, this exploratory descriptive study aims to describe the rate of unintended pregnancies, report on postpartum contraception management at discharge, identify women’s use and knowledge of prenatal/pregnancy vitamin supplementation, and identify modifiable lifestyle risks in pregnant women with severe mental illnesses attending for pregnancy care at a maternity hospital.

## Material and methods

This is a descriptive study of a sample of 38 pregnant women with a diagnosed severe mental illness referred for pregnancy care at a multidisciplinary antenatal clinic from May 2018 to June 2019 in Australia. The data presented here were from findings of the cross-sectional survey and prospective pregnancy data collection and was part of a prospective mixed-methods study undertaken on care, nutrition and lifestyle risk factors in women with SMI [17] and approved by the Hospital Ethics Research Committee (RGS0000000532). Informed written consent was obtained from all participants.

In total, 52 women received care through the study period, with exclusion of those with reduced capacity to consent due to being involuntary patients under the Mental Health Act and those with late referral for antenatal care in the third trimester of pregnancy. Seven women declined, resulting in an overall 84% participation rate. Women were recruited sequentially on referral and given a patient ID (*P1 − 38*). Pregnancy data were recorded on age, parity, smoking status prior to pregnancy and at booking visit, alcohol and substance use, body mass index (BMI) at time of booking, psychiatric diagnosis, psychotropic medication use, comorbid medical conditions, blood parameters of anaemia (Hb <110 g/L in pregnancy), iron deficiency (ferritin <30 ng/mL), active B12 and vitamin D; these were collected by the obstetric and psychiatric medical team. Discharge information on contraception recommendations were noted from the postnatal medical record summary.

Additionally, a paper-based survey was self-administered, either at the women’s first or second antenatal visit (around 16 weeks gestation), and collected data on country of birth, education, intended pregnancy or not, vitamin supplements use prenatally and during pregnancy, and information on knowledge and sources of knowledge about vitamin supplementation.

Descriptive statistics (proportion, mean and standard deviation) were used to describe the sample, with analysis of quantitative data undertaken in SPSS 24. Rates for unintended pregnancy and prepregnancy folic acid use were reported for women with a diagnosis of bipolar disorder and schizophrenia, with data from other psychiatric diagnoses excluded. Statistical comparison was not made due to the underpowered sample size.

## Results

The study sample (*n* = 38) consisted of women with a primary diagnosis of bipolar disorder (*n* = 21), schizophrenia and schizoaffective disorder (*n* = 16), and one woman with a severe nonpsychotic complex post-traumatic stress disorder. Mean gestation at first hospital antenatal booking visit was 16.6 weeks (SD 3.7). Demographic, medical and lifestyle comorbidities are reported in Table 1.

**Table 1.** Demographics, medical and lifestyle comorbidities at first antenatal booking visit for women with SMI.

Variable	Number (%), *n* = 38
Age mean (SD)	31 (5.1)
Australian born	27 (71%)
Highest level of education	
Not completed year 12	7 (18.4%)
Completed year 12	5 (13.2%)
Technical/certificate course	13 (34.2%)
Tertiary	13 (34.2%)
Unintended pregnancy	16 (42%)
Bipolar	7 (33%)
Schizophrenia	9 (56%)
Obesity (BMI >30)	13 (35.1%)
Cigarette smokers prenatally	12 (31.6%)
Continued smokers in pregnancy	8 (21.1%)
Quit smoking during pregnancy	4 (10.5%)
Past history of substance use	18 (47.4%)
Present use of substance	2 (5.3%)
Pre-existing medical conditions	
Asthma	7 (18.4%)
Thyroid disorders	7 (18.4%)
Haematological conditions	5 (13.2%)
Neurological conditions	2 (5.3%)
Chronic pain disorders	2 (5.3%)
Previous gastric sleeve	2 (5.3%)
Chronic hepatitis	1 (2.6%)

SD: standard deviation; BMI: body mass index.

Overall, unintended pregnancy occurred in 42% (*n* = 16) of women, with elevated rates at 56% for women with a diagnosis of schizophrenia. Within this sample, 13% (*n* = 5) women had a long acting reversible contraception (LARC) inserted prior to being discharged post-delivery, while 45% (*n* = 17) were discharged with no contraception or a documented plan, and left to discuss contraception with their GP at the 6-week postnatal check.

The mean BMI at booking visit was 29.6 (SD 6.5), with obesity rates at 35% (*n* = 13). Rates of cigarette smoking in pregnancy were 21% (*n* = 8). Of the women who continued to smoke during pregnancy, 62.5% had reduced their daily cigarette intake by their first antenatal booking visit. Similarly, a past history of substance use prior to pregnancy was reported in almost half, but this was reduced markedly with only two women continuing to use cannabis in pregnancy.

Table 2 outlines the prescribed medication profile within the sample. The majority (*n* = 33, 87%) were prescribed and taking a psychotropic medication at their first hospital antenatal booking visit. Two or more psychotropic medications were prescribed for the management of some of the participants’ mental health (*n* = 15, 39%).

**Table 2.** Psychotropic medication use at first antenatal booking visit.

Psychotropic medication	Number (%), *n* = 38
Mood stabilisers	15 (39.5%)
Lithium	8 (21%)
Lamotrigine	7 (18.4%)
Antipsychotic agent	29 (76.3%)
Quetiapine	12 (31.6%)
Olanzapine	7 (18.4%)
Aripiprazole	7 (18.4%
Clozapine	1 (2.6%)
Paliperidone	1 (2.6%)
Amisulpride	1 (2.6%)
Antidepressants	14 (36.8%)
Escitalopram	4 (10.5%)
Fluoxetine	3 (7.9%)
Sertraline	3 (7.9%)
Desvenlafaxine	2 (5.3%)
Duloxetine	1 (2.6%)
Mirtazapine	1 (2.6%)

Table 3 shows the use of prenatal and pregnancy vitamin supplementation and knowledge source. Overall 37% (*n* = 14) of women reported taking folic acid supplementation prior to pregnancy. In general, women’s responses in the survey seemed knowledgeable about the use of folic acid ‘beneficial for the baby’s development (*P*3)’, ‘…prevention of neural tube defects (*P*21)’ and ‘…reduces the risk of miscarriage (*P*33)’. Two women with previous gastric sleeve surgery recognised the need for higher dose folic acid in pregnancy (*P31* and *P35*), as did several of the women prescribed lamotrigine. However, only 38% (*n* = 5) prescribed a mood stabiliser medication used a higher dose folic acid formulation. Women with bipolar disorder were more likely to use pre-pregnancy folic acid than those with schizophrenia (52% compared to 25%, respectively).

**Table 3.** Prenatal and pregnancy vitamin supplementation use.

	Number (%), *n* = 38
Information on vitamin supplementation use	
Given information on vitamin supplementation	31 (81.6%)
Source of information	
General practitioners	28 (90.3%)
Midwives	8 (25.8%)
Friends, family or their own research	8 (25.8%)
Mental health practitioners	6 (19.4%)
Vitamin supplementation use prepregnancy (in rank order)	
Folic acid (including in pregnancy multivitamin)	14 (37%)
Additional Vitamin D	8 (23.5%)
Iron	5 (13.2%)
Fish oil	4 (10.5%)
Calcium	1 (2.6%)
Vitamin supplementation use in pregnancy (in rank order)	
Folic acid (including in pregnancy multivitamin)	30 (80%)
Iron	19 (50%)
Additional Vitamin D	15 (39.5%)
Fish oil	4 (10.5%)
Calcium	2 (5.3%)

Many women required extra supplementation during their pregnancy due to identified vitamin deficiencies. Rates of micronutrient deficiencies documented include iron deficiency (ferritin <30ng/mL) (*n* = 27, 73%), low vitamin *D* < 50 nmol/L (*n* = 13, 34%), and low active B12 levels (<35nmol/L) (*n* = 2, 5%).

## Discussion

### Statement of principal findings

Overall, 42% of women with SMI in this descriptive study had unintended pregnancies, with a greater proportion of women with a diagnosis of schizophrenia having an unintended pregnancy and a lower proportion using prenatal folic acid compared to women with other SMI. Despite these rates of unintended pregnancy and a heightened psychiatric risk profile, almost one half of women were discharged from hospital postnatally with no planned contraception, even though postnatal contraception is routinely discussed prior to discharge at the study hospital.

The overwhelming majority of women reported receiving their knowledge of the importance of prenatal/early pregnancy vitamin use from their GPs, but only 37% had used folic acid prior to pregnancy. Over a third of women prescribed mood stabiliser medication were on an appropriately higher dose of folic acid. Women in the study had high rates of coexisting medical comorbidities, and lifestyle comorbidities such as obesity and smoking. Vitamin deficiencies occurred frequently in this group of women in pregnancy, with iron deficiency and vitamin D being the most commonly reported.

### Strengths and weaknesses of the study

Given the low prevalence of severe mental illnesses such as bipolar disorder and schizophrenia in the community, and the barriers to recruiting these patients for mental health research [18], a strength of this study is the high participation rate of 84% achieved. Regardless, the sample size was underpowered for detailed statistical comparison. The study was undertaken in a single maternity hospital which may limit the generalisability of findings, however, this is offset by the clinic being a state-wide service for the pregnancy care of women with severe mental illnesses.

A further limitation with the study was that women who were under the Mental Health Act and lacking capacity to consent, were excluded from the research. A consequence of this sample bias might be that unintended pregnancy rates are underestimated and use of prenatal folic acid less than reported. In women with mental illnesses unhealthy lifestyle risk factors, such as increasing alcohol and illicit substance use, are associated with increased sexual activity and less contraception use [3].

### Findings in relation to other studies

Overall, we report unintended pregnancy rates in women with a severe mental illness diagnosis comparable with the general population [8], but women with schizophrenia appear more likely to indicate an unintended pregnancy than those with bipolar disorder. The need for effective contraception for women with mental illnesses, particularly those with schizophrenia, is important given their vulnerabilities, and ideally, this should occur early to avoid an unplanned pregnancy. There have been calls for community mental health care providers, psychiatrists and GPs to counsel women with regard to contraceptive choices alongside their mental health management [19]. As seen in this study the majority of women with SMI use psychotropic medication: a time for opportunistic discussion on reproductive planning could occur at the time of routine prescribing.

Another option for increasing use of more effective contraception, particularly in vulnerable groups, is its delivery in the immediate postpartum period [20]. However, within our sample, only 13% of women had a long acting reversible contraceptive (LARC) inserted prior to discharge postnatally, with almost a half of women left to discuss contraception with their GP at the 6-week postnatal check. Often this is too late, with barriers existing to care, including the need for follow up appointments for insertion of a LARC, lack of training and service access difficulties [21]. These, in combination with a heightened risk of mental illness relapse in the immediate postpartum period [22], have the potential to increase the risk of unintended pregnancy further. Addressing these issues in a primary care setting remains challenging although improvements in education and training and advocating on behalf of women with SMI to hospital systems to provide early and reliable contraception may be beneficial.

Unintended pregnancy, age, socioeconomic status, ethnicity, limited health literacy and poor adherence may limit use of supplement micronutrients in the preconception period [23,24]. Overall 37% of women with SMI in our study reported taking folic acid in the preconception period. We found that only one in three women prescribed a mood stabiliser used elevated doses of folic acid in the preconception period and only one in four with schizophrenia used any folic acid prepregnancy. In the general population, it is estimated that less than half of pregnant women take folic acid supplementation prenatally [23], but given the significant risk posed with medication use in women with SMI, a goal should be to improve prenatal use rates and prescribe an appropriate 5 mg dose for those with increased need [13–15].

Another important consideration is that perinatal nutritional deficiencies such as iron, folic acid and vitamin D may have significant implications for neurodevelopment, with research implying a greater risk for the development of schizophrenia in the offspring [25]. Even though our reported rate of vitamin D deficiency is comparable to estimated national prevalence rates [26], and the prevalence of iron deficiency is high in pregnancy with uncertainty surrounding population estimates, this is concerning given that these nutrients are important for fetal development. Additionally, low iron stores and vitamin D deficiency have been linked to maternal mental health issues, particularly depression [27,28]. Screening and replacement therapy either prior to pregnancy or in the early pregnancy period could potentially improve outcomes for women with mental illnesses and their babies.

Our study findings support the existing literature that shows women with a psychiatric diagnosis have increased coexistent medical and lifestyle comorbidities including obesity [5,6] which have capacity to be managed prior to pregnancy in the GP setting. Already, raised awareness between people with SMI and metabolic risk has led to improvements in preventative screening [29] within this population, and this awareness should be extended to include a range of physical health issues including those that are pregnancy related. Management of obesity with a 10% reduction in preconception BMI can reduce the adverse risks of preeclampsia, preterm birth and gestational diabetes mellitus (GDM) [30], all of which women with SMI are particularly at risk [5]. Seeking dietary advice early is recommended for excess weight management in women with SMI as they tend to make poorer food choices and consume high amounts of both sweet and processed snacks [17].

Women with SMI in this study were twice more likely to smoke cigarettes than the 10% of women in the general population who report smoking in pregnancy [31]. Despite population studies suggesting that many women stop smoking in early pregnancy [32], women with mental illnesses may struggle. However, it is encouraging to see in our findings that they appear motivated to reduce and/or stop when pregnant. Qualitative research suggests that people with SMI believe that smoking helps them to cope with their mental illness [33] but despite these beliefs smoking cessation has not been shown to cause any deterioration in mental health and has significant benefits. Smoking cessation should be encouraged by GPs with similar strategies used to those employed in people without mental illnesses [34].

### Meaning of the study

The majority of women with severe mental illnesses visits general practitioners and rely on them as a valuable source of information. GPs have many opportunities to improve the reproductive and prenatal planning in this group, whilst recognising their complex health needs. The findings and principles of care discussed here are also fundamental to managing pregnant women with common mental disorders such as depression and anxiety. Addressing the gaps in use of effective contraception and proactive reproductive planning discussions could reduce the risk of unintended pregnancy and its consequences. Weight reduction, as well as optimising medical and lifestyle comorbidities, advising on prepregnancy folic acid, including higher dose recommendations for those that require it, and providing early vitamin supplementation in those with recognised deficiencies, as well as liaising early with specialist psychiatric services may ultimately improve outcomes for these women and their babies.
